# The kConFab experience – 14 years of biobanking

**DOI:** 10.1186/1897-4287-10-S2-A95

**Published:** 2012-04-12

**Authors:** H Thorne, E Niedermayr, L Williams, A Willems-Jones, L Djandjgava, K Ioculano, C Osinski, kConFab research nurses

**Affiliations:** 1 kConFab, Research Department, Peter MacCallum Cancer Centre, St. Andrew’s Place, East Melbourne, VIC, 3002, Australia

## 

kConFab, the Australian/New Zealand consortium for research into families at high risk of breast and ovarian cancer, has completed collection & recruitment of 1,421 families during the past 14 years. Biological material, genetic, epidemiological, and psychosocial data are collected from affected and unaffected, female and male participants over the age of 18. This material is available to peer reviewed, ethically approved and funded research projects. At present, kConFab supplies material to 52 research projects world-wide.

The kConFab biological repository contains blood specimens from a total of 12,340 participants and 233 best friend controls. The standardized blood processing protocol produces plasma, non lymph, blood pellet and white blood cell fractions. White blood cells undergo EBV transformation which can be used by in functional assays or as a replacement source of DNA/RNA. To date, 1511 unique EBV cell line transformations are available.

As of July 2011, 97% of kConFab families have had genetic testing; identifying 35% of families with a pathogenic, large genomic rearrangement (LGR) or splice site mutation in either *BRCA1* or *BRCA2*. An additional 11% of families carry unclassified variants in *BRCA1* or *BRCA2*; with a further 0.7 % with mutations in the *ATM*, *CHEK2* or *TP53* genes. Of the 1,924 female participants who harbour the germline mutation, 66% are affected with breast or ovarian cancer.

kConFab has collected a total of 898 fresh tissue collections, including prophylactic mastectomy and oophorectomy specimens; and has a large collection of archival specimens. The tissue bank consists primarily of breast and ovarian tissue (tumour and normal), with a small proportion of other tissues. Tumour tissue comprises 27% of the bank. Following collection, a full research pathology review is conducted, wherein features such percentage tumour, normal epithelial, lymph and necrotic components are scored.

kConFab has constructed a total of 23 tissue microarrays (TMAs) (both sporadic and familial tumours) from our tissue bio bank. Where possible, tumour is matched to normal from the same archival block.

kConFab are currently working to supplement our glass slide archive with a digital slide repository. This will provide researchers with high resolution, high quality whole slide digital images for ease of transport, storage, review and analysis. Currently we have >1000 slides scanned for more than 600 participants.

The kConFab resource enables researchers to answer important questions relating to familial aspects of breast cancer. Information about the kConFab resource and the application process is available on the web site (http://www.kconfab.org).

